# Frequency of *Dirofilaria immitis* infection in blood donor dogs of the Rio de Janeiro state

**DOI:** 10.29374/2527-2179.bjvm002223

**Published:** 2023-10-11

**Authors:** Genilson Pereira Gonçalves, Suzane Gallardo Xavier, Nathália da Conceição Lima, Alexandre José Rodrigues Bendas

**Affiliations:** 1 Undergraduate in Veterinary Medicine, Instituto de Veterinária (IV), Universidade Federal Rural do Rio de Janeiro (UFRRJ). Seropédica, Seropédica, RJ, Brazil.; 2 Veterinarian, MSc. Autonomus, Niterói, RJ, Brazil; 3 Veterinarian, MSc. Autonomus, Rio de Janeiro, RJ, Brazil; 4 Veterinarian, DSc. Departamento de Medicina e Cirurgia Veterinária, IV, UFRRJ. Seropédica, Seropédica, RJ, Brazil.

**Keywords:** heartworm, microfilaremia, antigenemia, verme do coração, microfilaremia, antigenemia

## Abstract

Dirofilariasis, a parasitic disease caused by the nematode *Dirofilaria immitis*, commonly known as heartworm, primarily inhabits the pulmonary artery and right heart of dogs and other animals. The disease is transmitted through diptera, predominantly from the *Culex*, *Aedes*, and *Anopheles* genera. Dirofilariasis is cosmopolitan in nature, endemic in coastal regions and tropical climates. Factors such as temperature, humidity, vector density, and the presence of definitive hosts significantly contribute to the spread of this parasitic disease. In the state of Rio de Janeiro, a prevalence of 58.6% of *D. immitis* infected animals has been recorded in municipalities like Niterói. Given that blood transfusions are routine clinical procedures and blood bags are not always accurately evaluated, an investigation into *D. immitis* infection in blood donor dogs from the metropolitan region of Rio de Janeiro was conducted. A total of 1044 blood donor dog files from a blood bank in Niterói, RJ, collected from January 2019 to December 2022, were analyzed. These samples, originating from kennels in various municipalities in the Metropolitan Region, were tested for the presence of microfilariae through direct examination using tubes and microhematocrit evaluated in optical microscopy. Additionally, the search for antigens was conducted using the enzyme-linked immunosorbent assay technique. Out of the 1044 records evaluated, 17.8% (186/1044) were positive for heartworm infection, with 2% (21/1044) samples positive for microfilariae and 14.8% (154/1044) positive for *D. immitis* antigens. The high prevalence rate indicates that canine *D. immitis* infection remains prevalent in the state of Rio de Janeiro, necessitating effective guidelines for prescribing preventive medications by veterinarians and an increase in epidemiological surveillance in the region.

## Resumo

A dirofilariose é uma parasitose determinada pelo nematóide *Dirofilaria immitis*, que tem como habitat artéria pulmonar e coração direito de canídeos e outros animais, sendo por isso conhecido popularmente como verme do coração. A transmissão da parasitose ocorre por meio de dípteros principalmente dos gêneros *Culex, Aedes e Anopheles.* A dirofilariose é considerada cosmopolita, sendo endêmica em regiões litorâneas e de clima tropical. A temperatura, a umidade, a densidade de vetores e a presença de hospedeiros definitivos são considerados os principais fatores que contribuem para a propagação da parasitose. No estado do Rio de Janeiro, alguns municípios como Niterói, já foi registrada uma prevalência de 58.6% de animais infectados por D. immitis. Como as transfusões de sangue são procedimentos clínicos rotineiros e as bolsas de sangue nem sempre são avaliadas corretamente, decidiu-se investigar a infecção por D. immitis em cães doadores de sangue da região metropolitana do estado do Rio de Janeiro. Foram analisados 1044 prontuários de cães doadores de sangue de um banco de sangue localizado em Niterói, RJ, obtidos no período de janeiro de 2019 a dezembro de 2022. As amostras foram provenientes de canis localizados em diferentes municípios da Região Metropolitana, e testadas para presença de microfilárias por meio de exame direto utilizando-se tubos e microhematócrito avaliados em microscopia óptica e através da realização da pesquisa de antígenos utilizando-se a técnica de ELISA. Das 1044 fichas avaliadas, 17.8% (186/1044) das amostras foram positivas para infecção pelo verme do coração, sendo 2% (21/1044) positivas para presença de microfilárias e 14.75% (154/1044) positivas para presença de antígenos de *D. immitis*. A alta taxa de prevalência encontrada permite concluir que a infecção canina por D. immitis ainda apresenta alta prevalência no estado do Rio de Janeiro, exigindo orientações efetivas para a prescrição de medicamentos preventivos por médicos veterinários e aumento da vigilância epidemiológica na região.

## Introduction

Canine heartworm, a global affliction caused by the parasite *Dirofilaria immitis*, is typically found in regions with hot climates and environments conducive to the growth of culicid vector species (Dantas-Torres & Otranto, 2013). Coastal regions, which possess these characteristics, often report the highest incidence of this parasitic disease (Genchi et al., 2011). Dogs with microfilaremia are the primary source of parasitosis dispersion, as they often travel with their owners and can infect local vectors susceptible to the disease (Guerrero et al., 1992). A study by Labarthe et al. (2014) revealed a national prevalence of 23.1% in the Brazilian states examined. In the municipality of Niterói, this rate escalated to 58.6%. According to Silva et al. (2019), the prevalence of this parasitosis in the state of Rio de Janeiro was 24.1%.

In dogs, the impact of infection by this nematode is contingent upon several factors: the parasitic load, duration of infection, host immune response, and level of physical activity. Most patients remain asymptomatic (Ames & Atkins, 2020). However, when symptoms do manifest, the most common clinical signs include exercise intolerance (observed in approximately 46% of infected dogs), unproductive cough (42%), and loss of appetite (25%) (Polizopoulou et al., 2000). In extreme cases, the infection can lead to right congestive heart failure, syncope, vena cava syndrome, and even death (Atwell et al., 1988).

Infection of canines by *D. immitis* can be detected through the identification of microfilariae or antigens of adult parasites in blood samples (Batista et al., 2008). The investigation and identification of microfilariae are typically conducted using the Knott (1939) technique, as modified by Newton and Wright (1956), a method that is globally recognized (Rawlings, 1986; Soulsby, 1968). Antigen detection is accomplished through enzyme-linked immunosorbent assay (ELISA) or immunochromatographic methods, utilizing various commercial tests. These tests have demonstrated high specificity, with sensitivity contingent on the number of female parasites the host carries. Some tests are capable of detecting a single female *D. immitis* (Henry et al., 2018; Nelson et al., 2018).

Given the high incidence of dirofilariasis in Rio de Janeiro, this study aimed to document the infection rate of *D. immitis* among blood donor dogs. This was achieved by analyzing records from a blood bank situated in the Niterói/RJ municipality.

## Material and methods

### Characterization of the study area

The state of Rio de Janeiro, along with three other states, constitutes the Southeast region of Brazil. Characterized by a tropical climate, high temperatures, Atlantic Forest formations, and mangroves, it also boasts an extensive coastline along the Atlantic Ocean.

### Analysis of the files

We reviewed the records of canine blood donors, all over one year of age, whose samples were collected between January 2019 and December 2022. These samples were obtained from a private blood bank situated in Niterói, RJ.

### Processing of blood samples

Prior to blood donation, all dogs were assessed for *D. immitis* infection via microfilariae and *D. immitis* antigen investigations.

Microfilariae were identified through direct examination, utilizing a microhematocrit capillary tube and an optical microscope with a 100× magnification (De Carli, 2001). Antigen detection was performed using the ELISA technique with commercial kits (Snap™ 4Dx® - IDEXX), which identify the presence of antigens in female *D. immitis* older than 8 months.

The animal count and the percentage of positive cases were analyzed for each mesoregion of the State. These regions included North Fluminense, Northwest Fluminense, Baixada Fluminense, Metropolitan Region, Center Fluminense, and South Fluminense.

### Statistical analyses

The breed, age, sex, and region of residence for each parasite, along with the number of positive or negative samples, were recorded in a Microsoft Excel spreadsheet.

## Results

An analysis was conducted on 1044 records of donor dogs. The sample included 435 males (41.67%) and 609 females (58.33%), with ages ranging from 3 to 10 years and an average age of 5 years. The most commonly represented breeds were the American Bully (366/1044), Pitmonster (257/1044), Rottweiler (114/1044), and White Swiss Shepherd (67/1044).

The analysis of the records revealed that 17.71% (185/1044) of the samples tested positive for *D. immitis*. Out of the 185 diagnosed dogs, 111 were female (60%) and 74 were male (40%). In the antigen-only test, a total of 154 samples (154/185 - 83.24%) were positive. However, microfilariae were only detected in 21 samples (21/185-11.35%) upon direct examination. When considering both the presence of microfilariae and antigen detection, only 10 samples (10/185 - 5.41%) tested positive. The results obtained from the mesoregions are presented in Table 1.

**Table 1:** Canine *Dirofilaria immitis* infection diagnosis in blood donor dogs across Rio de Janeiro’s mesoregions: antigens and microfilariae investigation.

	N	Ag+	Mf+	Total+
North fluminense region	647	17.5% (113/647)	3.7% (24/647)	20% (131/647)*
Araruama	4	0% (0/4)	25% (1/4)	25% (1/4)
Barra de São João	18	27.8% (5/18)	0% (0/18)	27.8% (5/18)
Cabo frio	82	13.4% (11/82)	2.4% (2/82)	14.6% (12/82)*
Campos	166	12% (20/166)	6% (10/166)	17.5% (29/166)*
Iguaba Grande	10	30% (3/10)	0% (0/10)	30% (3/10)
Macaé	18	0% (0/18)	0% (0/18)	0% (0/18)
Maricá	246	22% (54/246)	4% (10/246)	24.7% (61/246)*
Rio das Ostras	54	31.5% (17/54)	1.8% (1/54)	31.5% (17/54)*
Saquarema	32	9.4% (3/32)	0% (0/32)	9.4% (3/32)
Cachoeiras de Macacu	17	0% (0/17)	0% (0/17)	0% (0/17)
Metropolitan region	316	14% (44/316)	2.2% (7/316)	15.2% (48/316)*
Itaboraí	28	7% (2/28)	0% (0/28)	7% (2/28)
Magé	11	18.2% (2/11)	9% (1/11)	27.3% (3/11)
Niterói	104	19.2% (20/104)	3.8% (4/104)	21.1% (22/104)*
Nova Iguaçu	37	5.4% (2/37)	0% (0/37)	5.4% (2/37)
Rio de Janeiro	10	10% (2/10)	0% (0/10)	10% (2/10)
São Gonçalo	92	14.1% (13/92)	2.2% (2/92)	15.2% (14/92)*
Tanguá	34	8.8% (3/34)	0% (0/34)	8.8% (3/34)
South fluminense region	37	5.4% (2/37)	0% (0/37)	5.4% (2/37)
Barra Mansa	17	11.8% (2/17)	0% (0/17)	11.8% (2/17)
Resende	20	0% (0/20)	0% (0/20)	0% (0/20)
Center fluminense region	44	11.4% (5/44)	0% (0/44)	11.4% (5/44)
Friburgo	7	0% (0/7)	0% (0/7)	0% (0/7)
Petrópolis	4	25% (1/4)	0% (0/4)	25% (1/4)
Teresópolis	33	12.1% (4/33)	0% (0/33)	12.1% (4/33)

* Presence of one or more samples testing positive for both examinations.

N: number of samples, Ag +: number of samples with antigen for *D. immitis*, Mf +: number of samples with microfilariae, Total +: number of samples that contain both microfilariae and the antigen for *D. immitis*.

**Figure 1:**
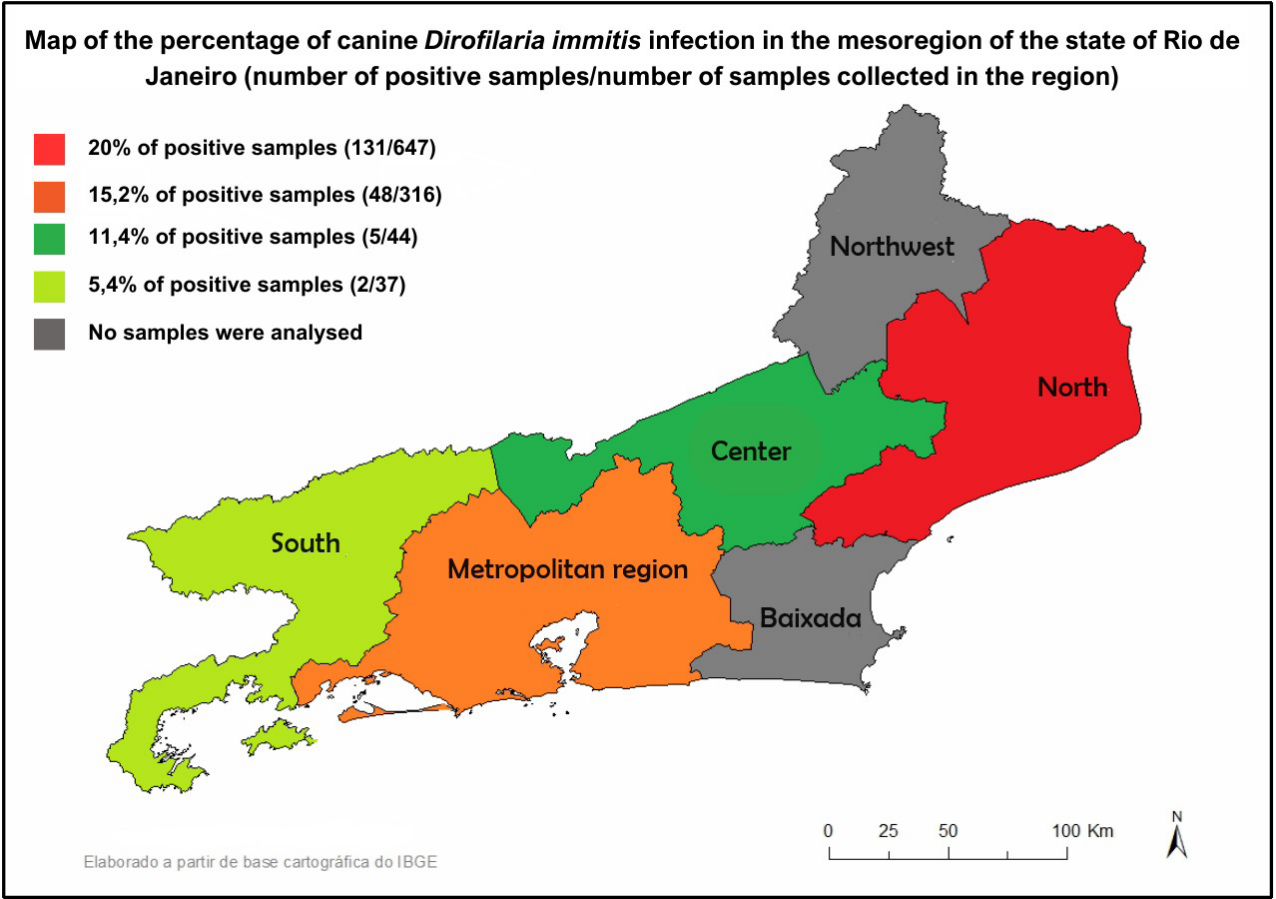
Rio de Janeiro State map: mesoregions and their canine *D. immitis* infection positive sample percentages.

Note. In the Rio de Janeiro region, positive animal cases were as follows: 20% (131/647) in the North, 15.2% (48/316) in the Metropolitan Region, 11.4% (5/44) in the Center, and 5.4% (2/37) in the South. Samples from the Northwest Fluminense and Baixada samples were not analyzed.

The animal count and the proportion of positive cases from each mesoregion of the State were analyzed. These regions included North Fluminense, Northwest Fluminense, Baixadas, Metropolitan Region, Center Fluminense, and South Fluminense (Figure 1).

## Discussion

The obtained results, indicating 17.71% of animals infected, align with the prevalence data reported by Silva et al. in 2019 (24.1%) for the state of Rio de Janeiro. This likely stems from the climatic and environmental conditions within the state, which are conducive to the vector’s development. The high canine population, both domiciled and otherwise, also contributes to this prevalence (Klinge et al., 2006).

The higher prevalence of purebred animals may be attributed to the fact that the blood bag donors were dogs from commercial breeding kennels. This could also account for the high incidence in specific regions, contingent on the kennel’s location.

The higher percentage of animals diagnosed through antigen research (83.24%) as opposed to microfilariae research (11.35%) can be attributed to the superior sensitivity of the ELISA technique (Das et al., 2023; Rawlings et al., 1982).

In the current study, approximately 88% of the animals were amicrofilaremic, a significant deviation from the literature, which typically reports amicrofilaremic infections in 30% of infected animals. This discrepancy may be attributed to the fact that the search for microfilariae in the microhematocrit capillary is not the gold standard for detecting microfilariae. Instead, concentration techniques, such as the Knott technique, should be employed (Das et al., 2023; Trancoso et al., 2020).

Nonetheless, 11.35% of the animals tested positive solely in the microfilariae test, indicating that the antigen test’s sensitivity is not absolute and could be influenced by the formation of immune complexes and the unique characteristics of the infection in each patient (Ferrer Montaño et al., 2002; Genchi et al., 2011; Vieira et al., 2014). Velasquez et al. (2014) highlighted the significance of preheating serum samples to diminish immune complexes for a more accurate diagnosis.

The presence of both circulating antigens and microfilariae in only 5.41% of the samples (10/185) underscores the necessity of conducting both tests, rather than relying on a single one, for accurate infection diagnosis (Nelson et al., 2018).

## Conclusions

The current study suggests that heartworm remains a significant disease in the state of Rio de Janeiro. Veterinarians in the region must maintain vigilance in accurately diagnosing the disease, employing at least two tests to confirm infection and intensifying preventive measures.
